# Integrative radiogenomics for virtual biopsy and treatment monitoring in ovarian cancer

**DOI:** 10.1186/s13244-020-00895-2

**Published:** 2020-08-17

**Authors:** Paula Martin-Gonzalez, Mireia Crispin-Ortuzar, Leonardo Rundo, Maria Delgado-Ortet, Marika Reinius, Lucian Beer, Ramona Woitek, Stephan Ursprung, Helen Addley, James D. Brenton, Florian Markowetz, Evis Sala

**Affiliations:** 1 Cancer Research UK Cambridge Institute, University of Cambridge, Cambridge, CB2 0RE UK; 2 Cancer Research UK Cambridge Centre, University of Cambridge, Cambridge, CB2 0RE UK; 3 Department of Radiology, University of Cambridge, Cambridge, CB2 0QQ UK; 4 Department of Biomedical Imaging and Image-guided Therapy, Medical University Vienna, 1090 Vienna, Austria; 5 Cambridge University Hospitals NHS Foundation Trust, Addenbrooke’s Hospital, Cambridge, CB2 0QQ UK

**Keywords:** Radiomics, Radiogenomics, Ovarian cancer, Tumour habitats, Virtual biopsies

## Abstract

**Background:**

Ovarian cancer survival rates have not changed in the last 20 years. The majority of cases are High-grade serous ovarian carcinomas (HGSOCs), which are typically diagnosed at an advanced stage with multiple metastatic lesions. Taking biopsies of all sites of disease is infeasible, which challenges the implementation of stratification tools based on molecular profiling.

**Main body:**

In this review, we describe how these challenges might be overcome by integrating quantitative features extracted from medical imaging with the analysis of paired genomic profiles, a combined approach called radiogenomics, to generate virtual biopsies. Radiomic studies have been used to model different imaging phenotypes, and some radiomic signatures have been associated with paired molecular profiles to monitor spatiotemporal changes in the heterogeneity of tumours. We describe different strategies to integrate radiogenomic information in a global and local manner, the latter by targeted sampling of tumour habitats, defined as regions with distinct radiomic phenotypes.

**Conclusion:**

Linking radiomics and biological correlates in a targeted manner could potentially improve the clinical management of ovarian cancer. Radiogenomic signatures could be used to monitor tumours during the course of therapy, offering additional information for clinical decision making. In summary, radiogenomics may pave the way to virtual biopsies and treatment monitoring tools for integrative tumour analysis.

## Key points

- Radiogenomics is the integration of radiomics and genomics and holds the potential to improve treatment response and outcome prediction in ovarian cancer.
- Creating virtual biopsies to overcome the limitations of invasive biopsies may be feasible due to recent technological advances that allow integration of imaging and molecular data.
- Radiogenomics studies that ensure reproducible, tissue-matched radiomic signatures in large prospective cohorts are needed for the clinical implementation to become a reality.

## Background

Ovarian cancer is the second most prevalent of gynaecological cancers [1]. While survival rates for most tumours have improved over the last decades, the 5-year survival rate of ovarian cancer has not changed since 1980 [2]. The majority of the ovarian tumours are high-grade serous ovarian carcinomas (HGSOCs) [2, 3] which are characterised by a high degree of heterogeneity that manifests on multiple levels. Biologically, HGSOC is a solid tumour driven by recurrent point mutations in TP53 and BRCA1/2 and by DNA copy number alterations [4–6]. Copy number changes arising from a variety of mutational processes, such as insertion, deletion or duplication of chromosomal fragments, are thought to allow for growing tissues to adapt to environmental selection forces [7]. Additionally, tumour microenvironment (TME) heterogeneity—i.e., the varying proportions of tumour, stroma and immune cells, as well as ascites components, such as cytokines and growth factors—is known to be a hallmark of HGSOC. The interplay of these factors is yet to be understood [8].

Clinically, HGSOC is frequently diagnosed as an advanced multi-site disease with lesions in the pelvis and peritoneal cavity [9]. The standard of care treatment is either primary debulking surgery followed by adjuvant chemotherapy, or neoadjuvant chemotherapy (NACT) followed by delayed primary surgery. HGSOC is particularly sensitive to platinum-based NACT due to defects in homologous recombination [10, 11]. However, treatment resistance develops in up to 90% of patients over time and is the main cause of death in HGSOC [12, 13]. The main hypothesis for the resistance mechanism is the presence of tumour cell clones with different genomic profiles that evolve to acquire treatment resistance [14–16]. Genomic changes are also linked to the heterogeneity of the TME, which may impose different selective pressures on clonal populations [17–20].

In order to improve the management of patients with HGSOC, new tools are required to enable earlier detection of resistance to chemotherapy and evidence-based therapeutic stratification. The main challenge for the implementation of such tools resides in accurately characterising the multi-scale complexity of the disease, across tumour regions and metastatic sites. So far, the main approach to evaluate intra- and inter-lesion heterogeneity has been multiple sampling. However, this approach is invasive, expensive and impractical and may still provide inadequate coverage of the disease depending on sample location [21].

Tools based on standard of care or advanced radiological imaging, both at diagnosis and throughout the course of therapy, may capture the spatiotemporal tumour complexity in a comprehensive and non-invasive manner [22]. This information can be useful to predict the probability of certain molecular profiles of interest, by obtaining virtual biopsies from radiological images, and allowing for real biopsies to be taken in a more informed way.

**Fig. 1:**
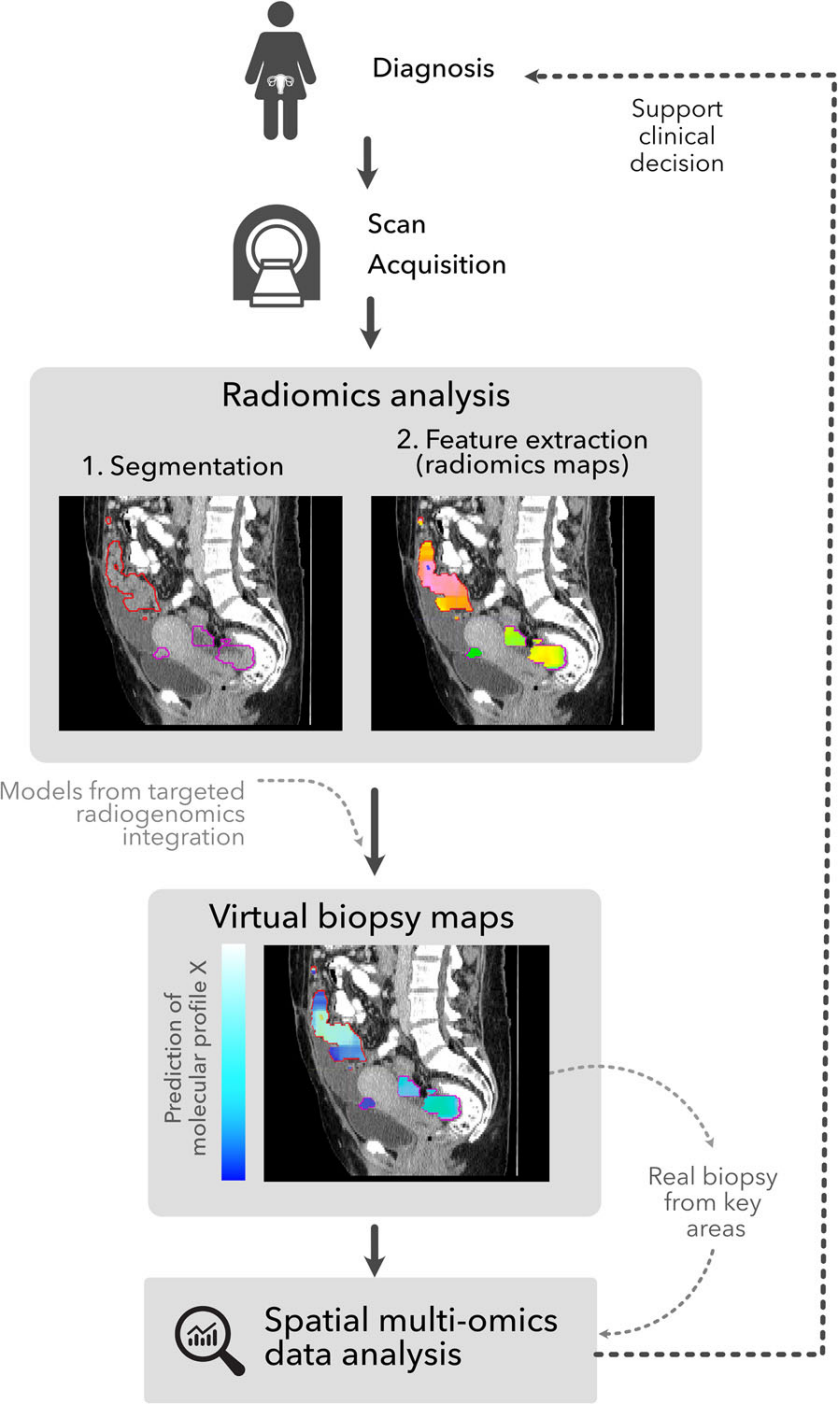
Overview of the workflow leading to the creation of virtual biopsy maps that can be used, together with real time biopsies from key areas, to inform clinical decisions. These virtual biopsy maps offer the possibility to be calculated at different stages of the clinical process—e.g., between chemotherapy cycles—to study the spatiotemporal variation of the molecular profiles

In the following sections, we first describe how radiomic analysis, the quantitative analysis of medical images, can be integrated with molecular information via radiogenomic studies. We then discuss the importance of tumour habitats, defined as regions with local distinct radiomic features, in this integrative process. We also outline technological advances to take habitat-specific samples to integrate molecular profiles and radiomics in a spatial manner. This may enable the creation of prediction maps for molecular profiles, so-called “virtual biopsies”. An overview of this process is presented in Fig. 1.

## Main text

### From radiomics to radiogenomics: comprehensive measures of tumour biology

Medical imaging, primarily computed tomography (CT), is crucial for diagnosing HGSOC, evaluating its extent and assessing treatment response [23, 24]. Although current routine evaluation is mostly semantic and qualitative [25], it has become widely accepted that mining images in a quantitative manner may add useful information for clinical decision-making [26].

The analysis of quantitative features extracted from imaging data on a large scale is commonly referred to as *radiomics* [27–29]. Radiomic studies exploit the fact that medical imaging encodes information of the underlying tissues and aims to define quantitative imaging biomarkers related to clinical endpoints [30]. The typical radiomics workflow consists of five steps performed on images of different modalities, usually CT, positron emission tomography (PET) or magnetic resonance imaging (MRI). The steps are (i) formulation of the clinical question and selecting the most appropriate type of imaging, (ii) tumour segmentation, (iii) image pre-processing, (iv) feature extraction from tumour regions (e.g., intensity, shape, volume and texture on original or filtered scans) and (v) predictive modelling of clinical endpoints through machine learning algorithms—including feature selection, model training and validation of the findings in internal or external datasets [31]. These steps and their limitations have been discussed in the existing literature [26, 28, 30–34]. More recently, radiomic analyses have also included deep learning methods, which have the advantage of being able to learn the most useful quantitative representations of the data by themselves, therefore bypassing the need for handcrafted features [35–37].

Radiomic studies have focused on understanding treatment response, recurrence and survival [38, 39]. Conceptually, the hypothesis underlying these analyses is that different imaging phenotypes capture the pathophysiology of the lesions [29]. The study of the association between radiomic and biological features, particularly genomics, is usually called *imaging genomics* or, more commonly, *radiogenomics* [40]. The field is still in its early stages, but initial results are promising.

Although limited radiogenomics literature is available in HGSOC, several studies showed a significant association of radiomics with relevant molecular profiles (Table 1). As discussed above, one of the most frequent mutations in HGSOC patients is in the BRCA1 or BRCA2 genes [49]. Patients with BRCA mutations tend to have better responses to chemotherapy due to the higher platinum sensitivity, and this improves their overall survival when compared to BRCA wild-type patients [50, 51]. Using features from CT in a dataset with 108 HGSOC patients, a higher likelihood of having mutant BRCA was found to be related with nodular pelvic disease and the presence of disease in the gastrohepatic ligament, whilst infiltrative pelvic disease, mesenteric presence and supradiaphragmatic lymphadenopathy were related with decreased likelihood of BRCA mutation [42]. Additionally, the same study reported the higher likelihood of incomplete resection in BRCA wild-type HGSOC in cases with mesenteric involvement and lymphadenopathy in supradiaphragmatic, as well as shorter PFS in both mutant and wild-type BRCA patients with suprarenal para-aortic regions and pelvic disease presence in the lesser sac and left upper quadrant and the mesenteric involvement relationship [42].

**Table 1:** Summary of radiogenomics papers focusing on High Grade Serous Ovarian Cancer (HGSOC). CLOVAR refers to the Classification of Ovarian Cancer proposed in [41]

Method	Ref.	Patients	Imaging	Level of association	Biological correlate	Results
Regular association	[42]	108	CT	Genomics	BRCA mutation	Nodular pelvic disease and the presence of pelvic disease in the gastrohepatic ligament associated with high likelihood of BRCA. Infiltrative pelvic disease, mesenteric presence and supradiaphragmatic lymphadenopathy were related with less likelihood of BRCA mutation.
	[43]	38	CT	Genomics	19q12 amplification	High inter lesion heterogeneity is associated with the amplification of 19q12.
	[44]	46	CT	Transcriptomics	CLOVAR classification	Peritoneal disease and mesenteric infiltration related with the mesenchymal subtype of the CLOVAR.
	[45]	92	CT	Transcriptomics	CLOVAR classification	Peritoneal involvement and presence of disease in the pelvis and ovary related with mesenchymal subtype.
	[46]	20	CT	Proteomics	Selected proteins	Intra- and inter-site heterogeneity associated with selected proteins involved in aminoacid metabolism.
	[47]	297	CT	Multilevel	DNA damage and stromal phenotype	CT-based radiomic signature with prognostic capacity positively associated with a stromal phenotype and negatively correlated with markers of DNA damage.
Targeted analysis	[48]	1	MRI	Multilevel	Histology and genomics	Different imaging habitats were related to different growth patterns when looking at the histopathological examination. Hypoxia and neovascularisation markers also differ between habitats. Using presence of somatic mutations and gene copy number variation, phylogenetic tree reconstruction showed that the habitats derive from different clones.

Looking also at the genomic level, a high-resolution genome-wide study of copy number variations in 118 ovarian cancer patients revealed the association of 19q12 amplification, which contains the cyclin E1 gene (CCNE1), with failure of primary treatment and worse survival [52]. CCNE1 is known to play a role in different processes of tumour cells, such as G1 to S phase transition, replication of DNA, apoptosis and chromosomal instability [53]. Using CT-based radiomic analysis of 38 HGSOC patients, Vargas and co-authors [43] reported that high variation of texture features between lesions—described as high inter lesion heterogeneity—was associated with the amplification of 19q12.

Using transcriptomic information from The Cancer Genome Atlas (TCGA) [54], four subtypes of HGSOC with prognostic relevance known as the CLOVAR Classification of Ovarian Cancer were defined—differentiated, immunoreactive, proliferative and mesenchymal [41]. The latter subtype, that is characterised by higher platinum resistance and worse prognosis, establishes the important role of stromal TME in HGSOC [55]. A semantic classification of CT scans based on the peritoneal disease and mesenteric infiltration at diagnosis was found to be associated with the mesenchymal subtype of the CLOVAR classification in 46 patients with HGSOC [44]. The importance of peritoneal involvement in the patients that fall under the mesenchymal subtype was further validated in a multisite cohort of 92 HGSOC patients in which 38 of the patients present in [44] were included [45]. It was also reported that the presence of disease in the pelvis and ovary is associated with the mesenchymal subtype [45].

The proteomic level relevance in HGSOC was shown in the capacity to predict recurrence and survival of the Protein-driven Index of Ovarian Carcinoma (PROVAR), defined using reverse-phase protein arrays in 412 patients from the TCGA [56]. To explore the associations of proteomics and radiomics, a cohort of 20 patients with HGSOC was used together with the expression of proteins involved in amino acid metabolism with highly correlated protein and transcript levels [46]. Imaging traits capturing intra- and inter- site heterogeneity from CT in a similar manner to [43] were found to be associated with selected proteins such as STXVP2, ASS1 and CBD [46].

Lastly, looking to associations with markers at different levels—genomic, transcriptomic, proteomic and histopathology—a CT-based radiomic signature obtained from 297 HGSOC patients was found to be positively associated with a stromal phenotype, correlated to poor prognosis in different tumour types including ovarian cancer and negatively correlated with markers of DNA damage [47].

Similar radiogenomic associations have also been reported for other cancer types and imaging modalities such as MRI (glioblastoma [57, 58], oligodendrogliomas [59] and breast cancer [60, 61]), CT (lung cancer [62], lung adenocarcinoma [63] and hepatocellular carcinoma [64]) and PET (non-small cell lung cancer [65, 66] and lymphoma [67]). Several reviews have already comprehensively compared these radiogenomic studies and their main limitations [68, 69].

**Fig. 2:**
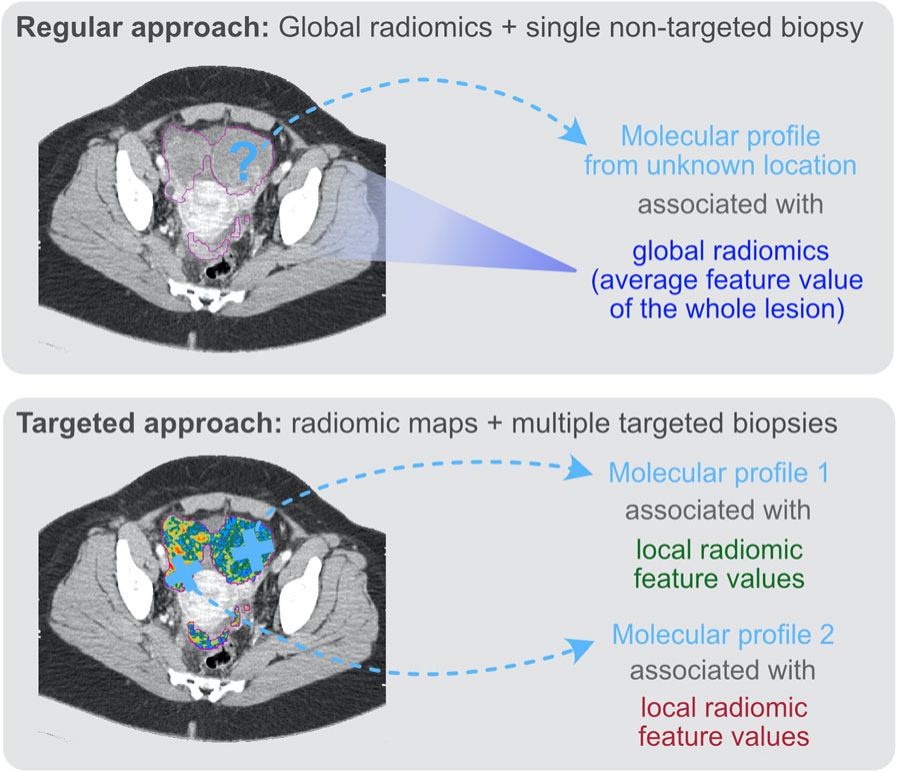
Comparison between the different approaches used for radiogenomic studies. Regular approaches usually extract a single value of each radiomic feature for the whole patient, obscuring radiomic habitats by assuming radiomic features are well mixed. This is then compared to the data obtained from a single biopsy from an unknown or approximate location. Targeted approaches overcome this limitation by utilising radiomic maps that convey local information for each radiomic feature. The molecular data to which the radiomic signatures are compared come from co-localised biopsies

These radiogenomic studies suggest important associations between radiomics and tumour biology, but it is important to note that they are generally based on datasets of limited size. Another key limitation is that these studies look for associations between radiomic features that are calculated for the whole lesion (or lesions) whilst the molecular profiling information is obtained from a single biopsy, sampling only a small part of the lesion or disease burden. This biopsy is often obtained by an image-guided procedure that provides anatomical information without capturing radiomic phenotypes (Fig. 2, approach 1). This strategy for guiding biopsies is the standard of care in most cancer types, but a single diagnostic biopsy cannot capture the complexity of whole tumours [70]. Likewise, the association of radiomic features with the molecular characteristics studied only in a single region or lesion may be hindering more specific and three-dimensional associations of imaging biomarkers with distinct molecular profiles.

### Habitat radiogenomics and targeted biopsies

**Fig. 3:**
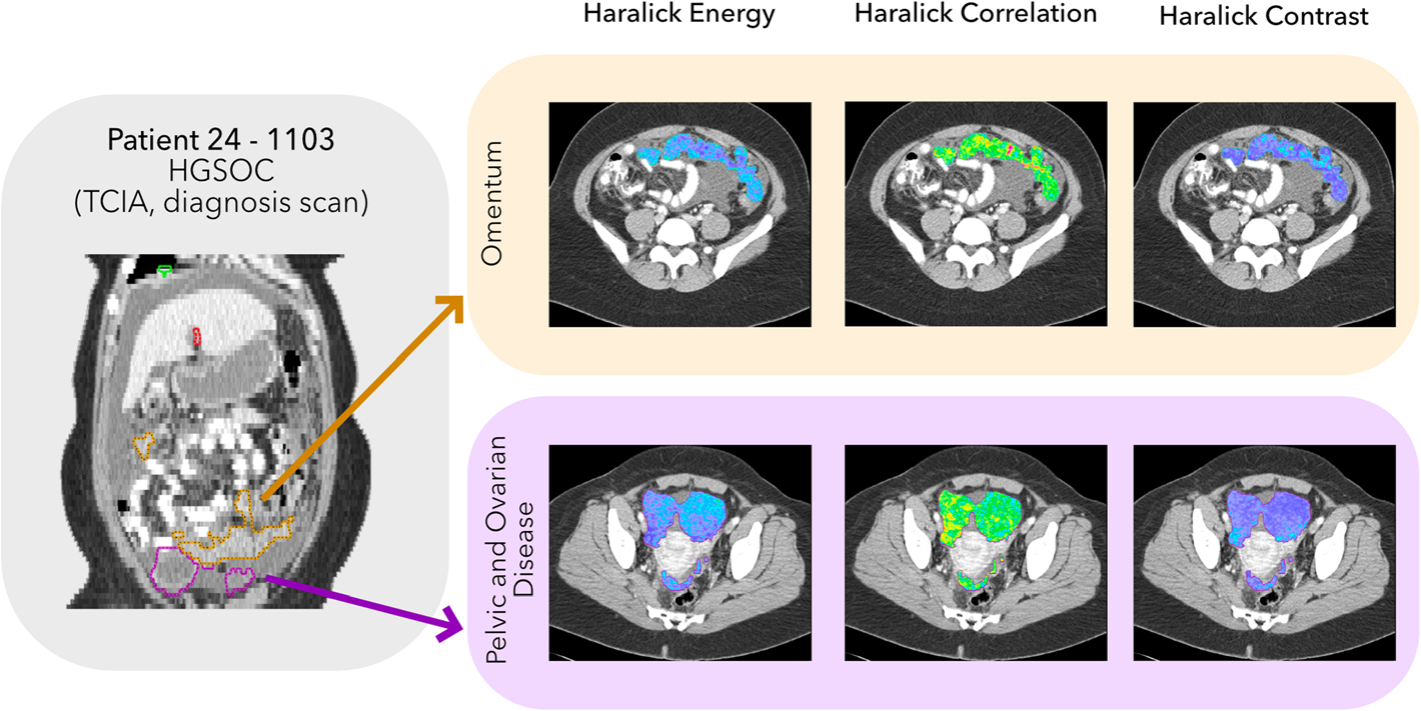
Example of texture analysis performed in a localised manner for omental and pelvic and ovarian disease (including the ovarian masses and peritoneal disease in the pouch of Douglas) of high-grade serous ovarian cancer (HGSOC) patient 24–1103 of The Cancer Imaging Archive (TCIA) repository to visualise the presence of distinct radiomic phenotypes in the same lesion. Texture maps were extracted using the Computational Environment for Radiotherapy Research (CERR) visualisation tool [71]. Haralick Energy, correlation and contrast look at different parameters of the Grey Level Co-occurrence Matrix (GLCM) used for texture analysis [72]. The brighter colours in the colourmaps refer to higher values of the parameters

Most radiomic analyses rely upon average quantitative features over the whole tumour volume, assuming that the imaging phenotype is the same across the lesion and between metastatic sites and therefore disregarding intratumoural heterogeneity [29]. Nevertheless, images often reveal heterogeneous patterns that can be appreciated quantitatively both in multiparametric functional imaging and in anatomic imaging through texture analysis (Fig. 3) [68]. Regions within a tumour that present a distinct imaging phenotype are called *habitats* [73, 74]. The presence of different habitats may indicate different underlying TMEs and tumour molecular profiles. Understanding the biological make-up of tumour habitats could therefore be key to elucidating the mechanisms of tumour evolution and resistance [75].

**Fig. 4:**
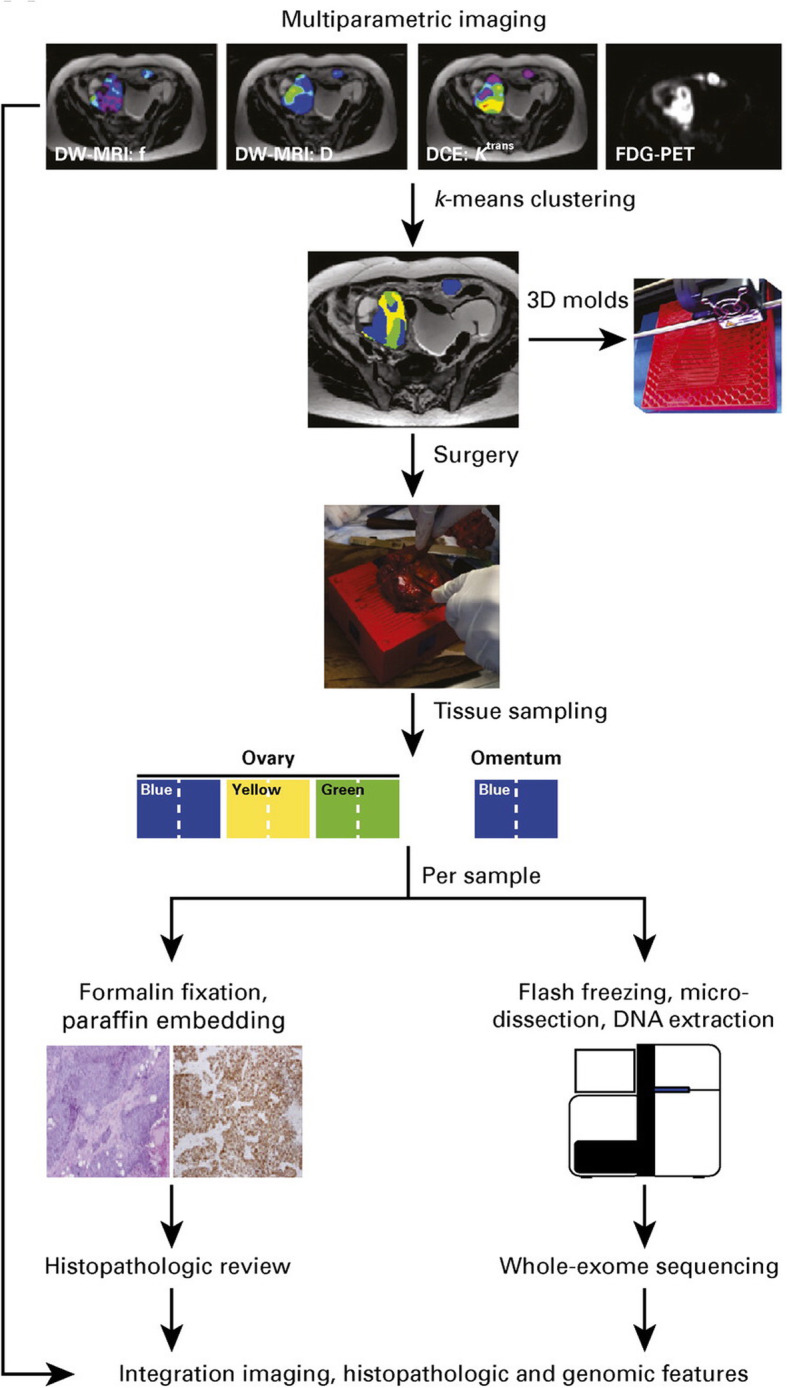
Workflow of the study performed in ovarian cancer where radiomic habitats were described by clustering different MRI sequences and fluorodeoxyglucose (FDG)-PET uptake values. Patient–specific 3D moulds were printed to sample each of the identified habitats. Histology and sequencing analysis were performed for each of the radiomic habitats. Reprinted with permission from [68]

To date, only a few studies have investigated the relationships between radiomic maps and spatially targeted molecular profiles to describe radiogenomic associations in a local manner. One of the key challenges presented by spatial associations is the accurate co-registration of images with tissue biopsies. Several methods have been proposed, and all of them are based on comparing tissue sections against imaging maps to guide the sampling. To achieve spatial overlap between imaging derived maps and tissue sections, most studies proceed by sectioning a resected specimen parallel to the slices of a pre-surgical scan. This sectioning process can be aided by 3D-printed moulds that ensure a correct alignment of the specimen with the MRI planes. Tissue sectioning can be facilitated further by slots integrated into the moulds that allow for specimen *trans*-sections parallel to the orientation of imaging slices for improved co-registration. In ovarian cancer, a 3D-printed mould for MRI-guided sampling was tested for a single patient, and areas with different imaging phenotypes obtained by combining perfusion, diffusion and metabolic information were shown to have different histology and genetic compositions [48] (Fig. 4, Table 1).

The mould-guided biopsy approach has recently gained popularity and has been used to investigate the association of radiomic features and histopathology phenotypes in different tumour types, such as prostate cancer [76–79], liver cancer [80] and renal cancer [81]. More recently, updates in the design of these moulds have been proposed to choose the preferred tissue sectioning angle, transforming the images and the corresponding maps [82]. This flexibility makes it easier for patient-specific moulds to be used in the clinical routine without disrupting the usual protocols.

The latest methods for targeting biopsies using radiomic maps have opened up new possibilities for associating radiomics and biological features in a localised manner. This is the first step toward comprehensive data integration and multiscale understanding of tumour complexity and behaviour. Nevertheless, most of the methods presented have two clear limitations: (i) very specialised technologies difficult to integrate in the clinical workflow and (ii) only possible after tumour resection, making it impossible to create multiscale models of tumours that track these associations longitudinally. Some of the most recent 3D mould technologies, such as the one proposed in [82], are designed to address the issue of clinical integration and may therefore also provide the key to increasing dataset size.

Accurately, correlating in vivo biopsies with distinct radiomic phenotypes is a different challenge for which fewer solutions exist [22]. A proof of concept based on MRI/US fusion was recently proposed in [83].

### The future of radiogenomics: challenges and open questions

Radiogenomics has emerged as a branch of radiomics that aims to explore associations between imaging phenotypes and biological correlates. Indeed, several studies have been published in different cancer types that support these interrelationships. When spatially co-localised, these studies could facilitate the spatial association of imaging phenotypes and biological pathways, leading to the creation of virtual biopsy maps.

This type of technology is needed in the setting of ovarian cancer to understand tumour evolution and apply this knowledge to clinical management. Detailed knowledge of the molecular tumour profile will enable a more personalised targeted therapy. Current analysis already shows the power of studying the radiogenomics associations in ovarian cancer (Table 1). The design and validation of custom 3D printed moulds to allow radiogenomics information to be integrated is a major key step toward this goal, especially in the surgical scenario. Nevertheless, the design of targeted biopsy methods that can be applied in the clinical setting in patients with HGSOC—using standard of care CT for example—would allow us to track these radiomic changes longitudinally during therapy and ultimately serve as virtual biopsy surrogates to inform clinical decisions.

There are some challenges that need to be carefully addressed before virtual biopsy methods can be integrated into the clinical practice. First, the reproducibility of radiomic features needs to be ensured. The calculation of different features varies between packages, and the numerical values are affected by scan acquisition and reconstruction parameters [33, 84]. Several strategies are in place to address these barriers and to ensure method robustness [38, 85, 86]. Second, the cohort size and high dimensionality of the multi-omics datasets to be integrated with the radiology information should be considered in the design of these studies to uncover meaningful associations with significant statistical power. Third, most of the studies on this subject are performed in a retrospective setting. The exploration of these associations in larger and prospective settings is mandatory for clinical translation. Finally, to ensure that these techniques can be utilised routinely in the clinic, targeted sampling methods need to integrate smoothly with clinical practice. For surgical biopsies, this will require robust and automated computational frameworks for 3D-printed mould production. For in vivo biopsies, one of the key developments will be a framework that allows radiologists to visualise radiomic maps in real time as they obtain the biopsy.

## Conclusion

Targeted radiogenomics could become a powerful addition to the integrative analysis of tumour biology and, thus, a valuable tool in the clinical management of HGSOC patients by predicting molecular profiles from standard of care CT scans. These virtual biopsies could revolutionise the current clinical practice in ovarian cancer, enable the development of biomarkers for treatment selection and detect treatment resistance early during the course of therapy.

## Abbreviations

CT: Computed tomography
EGFR: Epithelial growth factor receptor
HGSOC: High-grade serous ovarian carcinoma
MRI: Magnetic resonance imaging
NACT: Neoadjuvant chemotherapy
PET: Positron emission tomography
SUV: Standardised uptake value
TME: Tumour microenvironment
